# Resistance to deltamethrin and fenitrothion in dubas bug, *Ommatissus lybicus* de Bergevin (Homoptera: Tropiduchidae) and possible biochemical mechanisms

**DOI:** 10.1038/s41598-020-70150-7

**Published:** 2020-08-06

**Authors:** Rashad Rasool Khan, Thuwaini Hashil Abdullah Al-Ghafri, Salim Ali Humaid Al-Khatri, Ibtisam Salim Suliman Al-Mazidi, Fatma Gharib Al-Rawahi

**Affiliations:** 1 Plant Protection Research Center, Directorate General of Agriculture and Livestock Research, Ministry of Agriculture and Fisheries, Muscat, 121 Oman; 2 Department of Entomology, University of Agriculture, Faisalabad, 38000 Pakistan

**Keywords:** Entomology, Zoology

## Abstract

Environmental pollution, ill-effects on human health, insecticide resistance development and insect pest resurgence are some serious problems that may arise due to excessive chemical spraying for pest control. Despite of heavy aerial and surface insecticide spraying, incomplete control of *Ommatissus lybicus* de Bergevin 1930 (Homoptera: Tropiduchidae) is reported in Oman every year, which requires investigation of insecticides resistance in pest. Fifteen populations of *O. lybicus*, collected from diverse vicinities were exposed along with a deltamethrin-selected (DEL-SEL) and lab-susceptible (LAB-SUS) strain to deltamethrin and fenitrothion insecticides in bioassay tests for estimation of their resistance status. All the field populations of *O. lybicus*, exhibited minor (RR = 3–5-folds) to low (RR = 5–10-folds) levels of resistance to deltamethrin, however, two out fifteen populations collected from Al-Hajir and Sint were found susceptible against fenitrothion (RR < 3-folds). Enzyme assays were conducted to detect the activities of cytochrome p-450-reductase (CPR), glutathione s-transferase (GST) and acetylcholinesterase (AChE) in the field collected, DEL-SEL and LAB-SUS strains of *O. lybicus*. Results revealed significantly increased activities of all enzymes in the field collected as well as DEL-SEL strains of *O. lybicus* when compared with LAB-SUS strains.

## Introduction

Over reliance on chemicals for insect control can result in insecticide resistance development in crop pests and human disease vectors. Excessive and overuse of chemical pesticides by the farming community as well as pest exterminators may give rise to certain problems especially when pesticide resistance is developed in insect pests. Regardless of insecticide resistance development, the over and repeated use of pesticides may result in pest resurgence, environmental pollution, side effects on non-target organisms and health risks to consumers^1–5^. Alternate pest management tactics, judicious usage of insecticides, synthesis and development of biorational chemicals and insecticide resistance management programs can serve as the most appropriate solution to all these issues^6^.

Dubas bug, *Ommatissus lybicus* de Bergevin 1930 (Homoptera: Tropiduchidae), is demonstrated as the serious pest of date palm in Oman and causes economic losses by reducing the production of quality date fruits^7–10^. The pest records are also reported from Saudi Arabia, Egypt, Iraq, Israel, Yemen, Pakistan, Qatar, United Arab Emirates (UAE) other than Oman^11–14^. The higher *O. lybicus* populations can damage the date palms to greater extent and can make 25–50% crop losses^15^. Scientists describing the impacts of climate change on infestation of *O. lybicus* has declared northern Oman at the edge of risk and stated that it will bear sever pest influxes in future owing to ecological aptness^7^.

The plant protection research center in the Ministry of Agriculture and Fisheries, Oman applies insecticides including pyrethroids and organophosphates in alone and mixture combinations, through aerial and ground applications annually to regulate this obnoxious insect pest. A quantity of 523 tons of pesticides, costing 23 million dollars was sprayed for the control of this pest during 1993 to 2010^7^. Regardless of this heavy spraying, incomplete control of the pest has been reported by the farmers every year. The repeated and heavy spraying of date palm orchards with pesticides have resulted in resistance development in *O. lybicus*^16–18^. Since, *O. lybicus* adults are not able to fly longer distance, hence, the intensive application of pesticides can result in evolution of resistance in such populations^19^. Laboratory and field reports on toxicities and efficiencies of insecticides including neonicotinoids, organophosphates, pyrethroids and botanicals etc., against *O. lybicus* are published by various authors^18,20–27^. Fenitrothion, diazinon, cypermethrin and deltamethrin were reported more efficient and toxic against *O. lybicus* under laboratory and field situations^25,28^. A susceptibility survey of deltamethrin and fenitrothion against field collected populations of *O. lybicus* was conducted in Oman and the authors suggested the use of synergist (piperonyl butoxide, PBO) along with the two pesticides for efficient pest control and mitigating the resistance development in the pest^18^. If the resistance in such instance is checked effectively, the pesticide will remain affective for pest management.

The underlying mechanisms of insecticide resistance have attained significant consideration and became popular subject of research interest for many researchers around the globe^29,30^. In order to identify and study the resistance development mechanisms in certain insects, enzyme assays have been widely used as tools, because they are simple and rapid to perform, and sensitive to obtain results even with small insect samples^30^. The literature reports and proves that amplified levels of mixed function oxidases, aid in resistance development to carbamates, pyrethroids, organochlorines and organophosphates in certain insect pests^1,31–37^. Reports also describe the involvement of intensified esterases in mechanisms of resistance development against organophosphates, carbamates and pyrethroids^30,37–40^. Involvement and association of GST in the underlying mechanisms of resistance development against certain insecticides including organophosphates, organochlorines and pyrethroids has also been widely investigated and reviewed^30,36,41–44^. Furthermore, the AChE activities and role of insensitive and altered AChE in resistance to organophosphates and carbamates have been described in previous studies^30,32,33,45^.

As explained above, regardless of heavy insecticide spraying, severe infestations of *O. lybicus* are observed in date palm orchards in Oman. Development of resistance thus, has gained significant consideration as a reason for its control failures. No studies are documented to describe the underlying mechanisms of resistance development in *O. lybicus* against most commonly used insecticides. The present study was therefore planned to identify the underlying mechanism of resistance development in *O. lybicus*. Bioassay experiments were performed to appraise the status of insecticide resistance in different geographically isolated field collected *O. lybicus* populations against deltamethrin and fenitrothion (most often used insecticides against this pest in Oman). The enzyme analyses were further performed to detect the activities of CPR, GST and AChE in these field collected, DEL-SEL and LAB-SUS strains of *O. lybicus*. The findings reported in this study will provide baseline evidence about the current resistance status of the pest and the biochemical basis as a mechanism of resistance development for devising an affective and useful management strategy.

## Results

### Reference line susceptibility

The mortality scores of the laboratory reared nymphs of *O. lybicus* LAB-SUS strain were used as reference values for estimation of resistance and the corresponding LD_50_ values were used to calculate the resistance ratios (RRs) in the field collected populations. Both insecticides (deltamethrin and fenitrothion) showed highest toxicities to the LAB-SUS reference strain by displaying lowest LD_50_ values of 0.108 and 0.009 ppm, respectively. In comparison with the reference strain, neither the 95% CI values of field populations overlapped, nor the resistance ratios (RRs) contained 1 (Tables 1 and 2).

**Table 1:** Toxicity of deltamethrin in various field populations of *Ommatissus lybicus* under laboratory conditions.

Strain	n*	LD_50_**(95% CI***)	Slope (S.E)	χ^2^	df	P	RR**** (95% CI)
Falaj Al-Maragha	150	0.354 (0.296–0.383)	1.78 (0.17)	6.11	3	0.94	3.28 (2.68–3.91)^+^
Al-Hajir	150	0.466(0.409–0.516)	1.24 (0.15)	6.80	3	0.89	4.31 (3.56–5.15)^+^
Al-Wasil	150	0.348(0.302–0.401)	1.93 (0.17)	9.95	3	0.81	3.22 (2.77–3.86)^+^
Al-Kharma	150	0.502(0.442–0.578)	1.45 (0.16)	1.93	3	0.67	4.65 (4.19–5.35)^+^
Miss	150	0.508(0.457–0.589)	1.19(0.14)	9.67	3	0.54	4.70 (3.99–5.29)^+^
Ghiyazah	150	0.599 (0.527–0.684)	1.49(0.17)	9.79	3	0.56	5.54 (4.84–6.32)^+^
Al-Ghabbi	150	0.779 (0.708–0.881)	0.90(0.14)	0.18	3	0.69	7.21 (6.33–8.17)^+^
Al-Ruddah	150	0.790 (0.715–0.871)	0.99(0.14)	0.15	3	0.71	7.31 (6.85–8.02)^+^
Baa’d	150	0.881 (0.815–0.961)	1.36(0.15)	1.73	3	0.84	8.16 (7.20–9.41)^+^
Hail Al-Khanabisha	150	0.549 (0.481–0.603)	1.25 (0.14)	0.92	3	0.77	5.08 (4.24–6.06)^+^
Sint	150	0.398(0.320–0.471)	1.93 (0.17)	9.95	3	0.81	3.68 (2.77–4.66)^+^
Sayyah	150	0.502(0.412–0.608)	1.45 (0.19)	1.93	3	0.67	4.65 (3.89–5.45)^+^
Al-Hajar	150	0.408 (0.327–0.480)	1.19 (0.14)	9.67	3	0.54	3.78(3.19–4.69)^+^
Al-Waqba	150	0.399 (0.337–0.464)	1.49(0.17)	9.79	3	0.56	3.69 (3.04–4.22)^+^
Al-Mudhairib	150	0.679(0.608–0.781)	0.90 (0.14)	0.18	3	0.69	6.28 (5.33–7.37)^+^
DEL-SEL	150	0.795 (0.711–0.886)	1.50 (0.19)	0.85	3	0.42	7.36 (6.58–8.40)^+^
LAB-SUS (Ref. Strain)	150	0.108 (0.075–0.154)	0.99 (0.19)	0.70	3	0.68	1.0

*n = number of test insects, **LD_50_ = median lethal concentration (ppm), ***CI = confidence intervals, ****RR = resistance ratio, and +  = significantly different from the LAB-SUS (Reference) strain (95% CIs of RR didn’t include 1).

**Table 2:** Toxicity of fenitrothion in various field populations of *Ommatissus lybicus* under laboratory conditions.

Strain	n*	LD_50_** (95% CI***)	Slope (S.E)	χ^2^	df	P	RR**** (95% CI)
Falaj Al-Maragha	150	0.030 (0.017–0.047)	1.42 (0.17)	3.25	3	0.49	3.33 (2.91–3.94)^+^
Al-Hajir	150	0.044 (0.028–0.064)	1.29 (0.19)	2.19	3	0.41	4.89 (3.53–6.14)^+^
Al-Wasil	150	0.029 (0.011–0.047)	1.18 (0.16)	3.11	3	0.56	3.22 (2.63–3.84)^+^
Al-Kharma	150	0.039 (0.021–0.061)	1.19 (0.21)	3.68	3	0.49	4.33 (3.42–4.91)^+^
Miss	150	0.043 (0.031–0.057)	1.81 (0.19)	2.65	3	0.41	4.78 (4.03–5.63)^+^
Ghiyazah	150	0.046(0.027–0.067)	1.13 (0.21)	3.49	3	0.46	5.11(4.48–5.96)^+^
Al-Ghabbi	150	0.046(0.031–0.059)	1.13 (0.15)	4.32	3	0.19	5.11 (4.42–5.87)^+^
Al-Ruddah	150	0.051(0.037–0.066)	1.49 (0.17)	2.19	3	0.27	5.67 (5.12–6.51)^+^
Baa’d	150	0.065 (0.051–0.082)	1.69 (0.21)	3.89	3	0.19	7.22 (6.12–8.51)^+^
Hail Al-Khanabisha	150	0.053 (0.041–0.086)	1.17 (0.19)	1.91	3	0.21	5.89 (5.39–6.44)^+^
Sint	150	0.019 (0.009–0.036)	1.96 (0.23)	4.01	3	0.19	2.11 (1.53–2.91)^+^
Sayyah	150	0.035 (0.016–0.049)	1.71 (0.19)	4.22	3	0.11	3.89 (3.28–4.45)^+^
Al-Hajar	150	0.025 (0.014–0.039)	1.43 (0.19)	3.19	3	0.66	2.78 (2.12–3.31)^+^
Al-Waqba	150	0.029 (0.007–0.035)	1.52 (0.15)	2.96	3	0.37	3.22 (2.62–3.81)^+^
Al-Mudhairib	150	0.040 (0.052–0.094)	1.91 (0.17)	2.16	3	0.33	4.44 (4.09–4.86)^+^
DEL-SEL	150	0.039 (0.019–0.063)	1.28 (0.19)	3.97	3	0.58	4.33 (3.91–4.82)^+^
LAB-SUS (Ref. Strain)	150	0.009 (0.003–0.013)	0.99 (0.14)	1.84	3	0.39	1.0

*n = number of test insects, **LD_50_ = median lethal concentration (ppm), ***CI = confidence intervals, ****RR = resistance ratio, and +  = significantly different from the LAB-SUS (Reference) strain (95% CIs of RR didn’t include 1).

### Deltamethrin resistance in *O. lybicus* field populations

The entire field populations showed some resistance (minor to low levels) against deltamethrin by displaying resistance ratios between 3–10-folds (Table 1). Highest resistance ratio (8.16-fold) was determined in the insects collected from Baa’d with LD_50_ value of 0.881 ppm and exhibited a low level of resistance against deltamethrin. The insects collected from Al-Ruddah, Al-Ghabbi, Al-Mudhairib, Ghiyazah and Hail Al-Khanabisha also exhibited low levels of resistance against deltamethrin with resistance ratios ranging from 5.08 to 8.16-folds. The *O. lybicus* strain selected with deltamethrin also displayed a low level of resistance (RR = 7.36-folds) when tested with deltamethrin, with LD_50_ value of 0.795 ppm. Other populations collected from Miss, Al-Kharma, Sayyah, Al-hajir, Al-Hajar, Al-Waqba, Sint, Falaj Al-Maragha and Al-Wasil exhibited minor levels of resistance against deltamethrin and displayed resistance ratios between 3.22 to 4.70-folds. Comparison of the toxicity values of deltamethrin against field collected populations and LAB-SUS strain demonstrates that no field strain was found susceptible against deltamethrin.

### Fenitrothion resistance in *O. lybicus* field populations

Five out of fifteen *O. lybicus* field populations unveiled low levels of resistance (RR = 5–10-folds), whereas, eight populations displayed minor levels (RR = 3–5-folds), when the representative toxicity values were compared with those of the LAB-SUS strain against fenitrothion (Table 2). Highest level of resistance (7.22-folds) was determined in the insects collected from Baa’d with LD_50_ value of 0.065 ppm. The same strain also exhibited a low level of resistance against deltamethrin. Insects collected from Hail Al-Khanabisha, Al-Ruddah, Al-Ghabbi and Ghiyazah also displayed low levels of resistance against fenitrothion with resistance ratios ranging between 5.11 and 5.89-folds. Minor levels of resistance (RR = 3–5-folds) were estimated in the insects collected from Al-Hajir, Miss, Al-Mudhairib, Al-Kharma, Sayyah, Falaj Al-Maragha, Al-Wasil and Al-Waqba. Insects collected from Al-Hajar and Sint were found susceptible by displaying low toxicity scores (RR < 3-folds) and LD_50_ values of 0.025 and 0.019 ppm, respectively. A minor level of resistance (RR = 4.33-folds) was also noted in the deltamethrin selected (DEL-SEL) strain with LD_50_ value of 0.039 ppm and exhibited some evidence of cross resistance.

### Enzyme activities in *O. lybicus* populations

The enzyme analyses were performed to detect the activities of CPR, GST and AChE in the field collected, DEL-SEL and LAB-SUS strain of *O. lybicus*. The analyses revealed significant increased activities of all assayed enzymes [CPR (DF = 16, F = 4,277.74, P = 0.000, R^2^ = 99.95); GST (DF = 16, F = 6.34, P = 0.000, R^2^ = 74.88); AChE (DF = 16, F = 146.79, P = 0.000, R^2^ = 98.57)] in the field collected as well as DEL-SEL strains of *O. lybicus* when compared with LAB-SUS strain (Table 3).

**Table 3:** Enzyme activities in various field populations of *Ommatissus lybicus.*

DB populations	CPR		GST		AChE	
	Activity ± SE (mU/mg)*	Ratio**	Activity ± SE (nmol/min/ml) *	Ratio**	Activity ± SE (U/L) *	Ratio**
Falaj Al-Maragha	216.59 ± 2.84d	2.86	68.92 ± 1.76abc	1.61	99.81 ± 2.44ghi	1.47
Al-Hajir	276.49 ± 3.25b	3.65	71.57 ± 3.04abc	1.67	86.98 ± 1.89ij	1.28
Al-Wasil	113.78 ± 3.65 k	1.50	68.26 ± 4.35abc	1.59	171.65 ± 3.89c	2.53
Al-Kharma	197.69 ± 2.31e	2.61	58.98 ± 2.65bcd	1.37	150.41 ± 3.57d	2.22
Miss	153.06 ± 1.99h	2.02	73.56 ± 2.30abc	1.71	190.52 ± 5.05bc	2.81
Ghiyazah	187.96 ± 2.72g	2.48	55.67 ± 2.29 cd	1.30	71.42 ± 1.77j	1.05
Al-Ghabbi	238.40 ± 2.81cd	3.14	69.58 ± 2.29abc	1.62	91.96 ± 2.78hi	1.36
Al-Ruddah	246.39 ± 2.35c	3.25	73.56 ± 5.94abc	1.71	123.06 ± 3.79ef	1.82
Baa’d	293.20 ± 2.32a	3.87	62.39 ± 5.26abcd	1.45	210.39 ± 6.65a	3.10
Hail Al-Khanabisha	155.27 ± 4.70h	2.05	55.00 ± 2.39 cd	1.28	205.44 ± 6.83ab	3.03
Sint	140.19 ± 2.48i	1.85	80.19 ± 1.33ab	1.87	110.43 ± 0.86fgh	1.63
Sayyah	167.59 ± 4.03gh	2.21	67.59 ± 2.30abc	1.57	145.21 ± 4.17d	2.14
Al-Hajar	132.18 ± 3.05j	1.74	58.98 ± 1.33bcd	1.37	114.42 ± 3.39 fg	1.69
Al-Waqba	83.95 ± 2.72 l	1.11	66.27 ± 2.39abc	1.54	198.06 ± 2.03ab	2.92
Al-Mudhairib	177.53 ± 4.35gh	2.34	77.53 ± 8.97ab	1.81	141.50 ± 1.28de	2.09
DEL-SEL	151.92 ± 3.47hi	2.00	82.84 ± 1.76a	1.93	139.54 ± 2.59de	2.06
LAB-SUS	75.82 ± 3.57 l		42.94 ± 1.33d		67.80 ± 1.65j	
Statistic summary	DF = 16, F = 4,277.74, P = 0.000, R^2^ = 99.95		DF = 16, F = 6.34, P = 0.000, R^2^ = 74.88		DF = 16, F = 146.79, P = 0.000, R^2^ = 98.57	

*Means sharing similar letters in a column do not differ significantly at 5% probability. **Enzyme activity ratios calculated with reference to the LAB-SUS (Reference) strain.

### Cytochrome P-450-Reductase (CPR) activity

Highest CPR activity (293.20 ± 2.32 mU/mg) was observed in the insects collected from Baa’d with an activity ratio of 3.87-folds in comparison with LAB-SUS strain (75.82 ± 3.57 mU/mg), which was followed by the insects collected from Al-Hajir (276.49 ± 3.25 mU/mg), Al-Ruddah (246.39 ± 2.35 mU/mg) and Al-Ghabbi (238.40 ± 2.81 mU/mg). Lowest enzyme (CPR) activities were observed in insects collected from Al-Waqba (83.95 ± 2.72 mU/mg) and Al-Wasil (113.78 ± 3.65 mU/mg) after the LAB-SUS strain and displayed enzyme activity ratios of 1.11 and 1.50-folds, respectively. Insects collected from Falaj Al-Maragha, Al-Kharma, Ghiyazah, Al-Mudhairib and Hail Al-Khanabisha exhibited a moderate activity of CPR with ratios of 2.86, 2.61, 2.48, 2.34 and 2.05-folds, respectively, when compared with enzyme activity of LAB-SUS *O. lybicus* population. The DEL-SEL strain also displayed an increased level of CPR activity (151.92 ± 3.47 mU/mg) with an activity ratio of 2-folds as compare to the LAB-SUS population.

### Glutathione s-transferase (GST) activity

The insects from DEL-SEL population displayed highest GST activity (82.84 ± 1.76 nmol/min/ml) with a GST activity ratio of 1.93-folds when compared with the enzyme activity of LAB-SUS *O. lybicus* population (42.94 ± 1.33 nmol/min/ml). Amongst the field collected populations, insects collected from Sint and Al-Mudhairib showed highest GST activities (80.19 ± 1.33 and 77.53 ± 8.97 nmol/min/ml, respectively). Whereas, minimum GST activities were observed in insects collected from Hail Al-Khanabisha (55.00 ± 2.39 nmol/min/ml), Ghiyazah (55.67 ± nmol/min/ml), Al-Kharma (58.98 ± 2.65 nmol/min/ml) and Al-Hajar (58.98 ± 1.33 nmol/min/ml), respectively. Insects collected from Miss, Al-Ruddah, Al-Hajir, Al-Ghabbi, Falaj Al-Maragha, Al-Wasil, Sayyah and Al-Waqba showed moderately higher levels of GST activities with activity ratios of 1.71, 1.71, 1.67, 1.62, 1.61, 1.59, 1.57 and 1.54-folds, respectively.

### Acetyl cholinesterase (AChE) activity

All the field collected populations and the DEL-SEL strain exhibited raised levels of AChE activity as compared to the LAB-SUS strain. Highest levels of AChE activity (210.39 ± 6.65 and 205.44 ± 6.83 U/L) were determined in the insects collected from Baa’d and Hail Al-Khanabisha, respectively, with enzyme activity ratios of 3.10 and 3.03-folds as compare to LAB-SUS *O. lybicus* strain (67.80 ± 1.65 U/L). Minimum AChE activity ratios (1.05, 1.28 and 1.47-folds) were determined in the populations collected from Ghiyazah (71.42 ± 1.77 U/L), Al-Hajir (86.98 ± 1.89 U/L) and Falaj Al-Maragha (99.81 ± 2.44 U/L), respectively. Insects collected from Al-Waqba, Miss and Al-Wasil also exhibited moderately higher AChE activities (198.06 ± 2.03, 190.52 ± 5.05 and 171.65 ± 3.89 U/L, respectively). The DEL-SEL strain also exhibited raised level of AChE activity (139.54 ± 2.59 U/L) with an enzyme activity ratio of 2.06-folds as compare to the LAB-SUS strain of *O. lybicus*.

## Discussion

In Sultanate of Oman, *O. lybicus* is reported to pose serious challenges for devising effective management strategies. Spraying of insecticides is the major pest control option which is carried out through area wide aerial applications using helicopters^10^ fitted with ULV spray equipment and through high pressure water pumps for ground and surface applications^12^. Emulsifiable concentrate (EC) and ultra-low volume formulations of organophosphorus and pyrethroid insecticides are more often used for ground and aerial applications, respectively^12,46,47^. Serious pest infestations in date palm orchards even after heavy aerial and surface spraying confer a challenge for the pest exterminators to devise suitable and environment-friendly pest management strategies^23,27,47,48^. Over and repeated use of pesticides may cause side effects to the non-target organisms and pose health risks to consumers as well as other organisms by contributing in environmental pollution^1–5^. Additionally, the injudicious pesticide spraying may result in insecticide resistance development and resurgence of the target and non-target pests. In order to avoid the unnecessary spraying of chemicals, it is necessary to investigate the status of insecticide resistance and the underlying mechanism for its mitigation. The research experiments were carried out to estimate the status of resistance in certain field collected populations of *O. lybicus* and understand the possible role of detoxifying enzymes in insecticide resistance development in the pest.

Results of our experiments demonstrated the indications of field evolved resistance in *O. lybicus* field populations against the tested formulations of insecticides with significantly different resistance levels in contrast with the reference (LAB-SUS) strain. Both insecticides (deltamethrin and fenitrothion) showed highest toxicities to the LAB-SUS reference strain, however, fenitrothion was more toxic amongst the tested insecticides. Susceptibility against deltamethrin was not reported in any field collected *O. lybicus* strain, however, insects collected from Al-Hajar and Sint were found susceptible against fenitrothion in contrast with the toxicity values of the insecticide against LAB-SUS strain. Six out of fifteen dubas bug field populations (Baa’d, Al-Ruddah, Al-Ghabbi, Al-Mudhairib, Ghiyazah and Hail Al-Khanabisha) exhibited low levels of resistance against deltamethrin with resistance ratios ranging from 5.08 to 8.16-folds. The *O. lybicus* strain selected with deltamethrin also displayed a low level of resistance (RR = 7.36-folds) when tested with deltamethrin, whereas remaining nine field populations collected from Miss, Al-Kharma, Sayyah, Al-hajir, Al-Hajar, Al-Waqba, Sint, Falaj Al-Maragha and Al-Wasil exhibited minor levels of resistance against deltamethrin.

Five out of fifteen *O. lybicus* field populations (Baa’d, Hail Al-Khanabisha, Al-Ruddah, Al-Ghabbi and Ghiyazah) exhibited low levels of resistance (RR = 5–10-folds), whereas, eight populations (Al-Hajir, Miss, Al-Mudhairib, Al-Kharma, Sayyah, Falaj Al-Maragha, Al-Wasil and Al-Waqba) displayed minor levels (RR = 3–5-folds), when evaluated with toxicity values of the LAB-SUS strain against fenitrothion. The insects collected from Baa’d showed a low level of resistance against both tested insecticides. Development of a minor level of resistance (RR = 4.33-folds) was also noted in the deltamethrin selected (DEL-SEL) strain and exhibited some evidence of cross resistance. We have reported previously that the field collected populations did not develop any moderate or higher level of resistance against deltamethrin and fenitrothion, but only one strain collected from Wadi Qari was reported to possess intermediate resistance level against fenitrothion^18^. The research presented in this study, however, does not report any field strain to possess intermediate or higher level of resistance against the tested insecticides. A possible reason for the slow development of resistance can be less number of generations in a year and a higher frequency of mutations is crucial for pesticide resistance development^49^.

Some field and laboratory studies were performed to appraise and evaluate the toxicity of certain chemicals against *O. lybicus* with the objective to pick the effective one for its management^21,23^. Pyrethroids (deltamethrin, bifenthrin and cypermethrin) were mostly reported as effective insecticides in controlling *O. lybicus* in the field studies^25,28^. However, only one report has surveyed and reported minor and low levels of resistance against deltamethrin and fenitrothion in *O. lybicus*^18^. We could not find any report describing the underlying mechanism of resistance development in *O. lybicus*. Elevated and amplified levels of mixed function oxidases in certain pest insects, are reported in literature to aid in resistance development to carbamates, pyrethroids, organochlorines and organophosphates^1,31–37^.

In our study, we also performed enzyme activity assays to estimate the activities of detoxifying enzymes viz., CPR, GST and AChE in the field collected, DEL-SEL and LAB-SUS strains of *O. lybicus* to determine their possible role in resistance development. The analyses revealed significant increased activities of all assayed enzymes in the field collected as well as DEL-SEL strains of *O. lybicus* when compared with LAB-SUS strains (DF = 16, P = 0.000). The involvement of CPR in resistance mechanism can be determined because a higher CPR activity was observed in the insects collected from Baa’d, Al-Hajir, Al-Ruddah and Al-Ghabbi. These populations were found resistant against both insecticides i-e deltamethrin (RR = 8.16-folds) and fenitrothion (RR = 7.22-folds). Lowest enzyme (CPR) activities were observed in insects collected from Al-Waqba and Al-Wasil after the LAB-SUS strain and displayed enzyme activity ratios of 1.11 and 1.50-folds, respectively. These populations exhibited minor levels of resistance against both insecticides. The insects from DEL-SEL population displayed highest GST activity whereas, amongst the field collected populations, insects collected from Sint and Al-Mudhairib showed highest GST activities. In our bioassay experiments, insects collected from these locations also exhibited minor to low levels of resistance. Insects collected from Miss, Al-Ruddah, Al-Hajir, Al-Ghabbi, Al-Wasil, Sayyah and Al-Waqba showed moderately higher levels of GST activities and were found with minor to low levels of resistance in bioassay experiments. Role of GST in resistance to certain insecticides including organophosphates, organochlorines and pyrethroids is also documented and described in many insects including mosquitoes and aphids^30,36,41–44^.

All the field collected populations and the DEL-SEL strain exhibited raised levels of AChE activity as compared to the LAB-SUS strain. The AChE activities and role of insensitive and altered AChE in resistance to organophosphates and carbamates have been described in previous studies^30–33,50^. Highest levels of AChE activity were determined in the insects collected from Baa’d and Hail Al-Khanabisha, respectively, with enzyme activity ratios of 3.10 and 3.03-folds as compare to LAB-SUS *O. lybicus* strain. Insects collected from Al-Waqba, Miss and Al-Wasil also exhibited moderately higher AChE activities (198.06 ± 2.03, 190.52 ± 5.05 and 171.65 ± 3.89 U/L, respectively). The raised level of AChE activity (139.54 ± 2.59 U/L) in DEL-SEL strain with an enzyme activity ratio of 2.06-folds describes its involvement in resistance development in field populations of *O. lybicus*.

Conclusively, the study reveals minor to low levels of resistance development in the field populations of *O. lybicus*. As a consequence of selection pressure under frequent and heavy spray regimes, the pest may develop even higher levels of resistance due to enhanced activities of detoxifying enzymes. Discontinuation of spraying with deltamethrin and fenitrothion and use of alternate chemical pesticides in rotation can help to mitigate the resistance development in *O. lybicus*. Further studies are also recommended to investigate more efficient and eco-friendly pesticides for inclusion in pest management program in Omani date palm orchards. Less reliance on conventional pesticides and use of biorational insecticides along with some alternate pest management strategies will help in insecticide resistance mitigation and reducing the environmental pollution.

## Materials and methods

All the bioassay and enzyme activity assay procedures were performed in the Bioassay Research Laboratory and Entomology Research Laboratory, Plant Protection Research Center, Directorate General of Agriculture and Livestock Research, Al-Rumais, Oman.

### Insect collection and rearing

**Figure 1:**
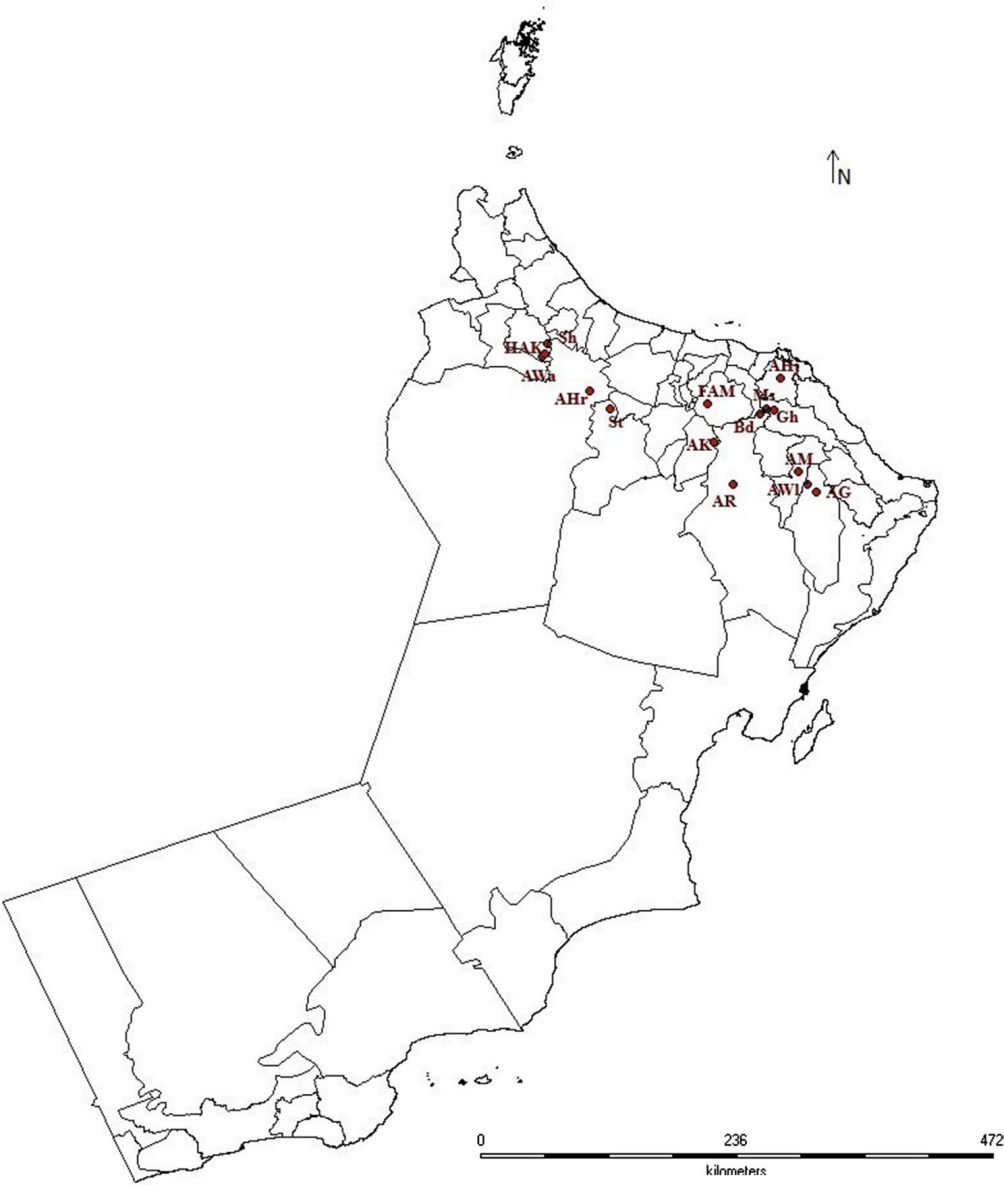
*Ommatissus lybicus* collection sites from sprayed date palm orchards in Oman.

Fifteen field populations of *O. lybicus*, were collected during 2019 after performing extensive surveys of date palm orchards located in different geographically isolated locations (Table 4, Fig. 1). The insects were collected from the date palm orchards having medium to high pest infestation and were shifted to the mesh cages (80 × 60 × 50 cm) containing potted date palm off-shoots, maintained under the laboratory conditions of 27 ± 2 °C, 70 ± 5% RH and 12L:12D photoperiod. A lab strain was used as a reference strain (LAB-SUS) which was collected in 2013 from unsprayed date palms and was maintained without exposing to any insecticide under lab conditions. However, some batches of insects from the LAB-SUS strain were used to select successively by exposing to sub-lethal doses of deltamethrin till seven generations^18^. The 13th generation of the same DEL-SEL strain was again tested for its resistance status in our current experiments.

**Table 4:** List of locations and insect infestation.

Location	Coordinates	Date of insect Collection	Insect Stage	Infest. Level
	Latitude; Longitude			
Falaj Al-Maragha (FAM)	23°10′42″N, 57°54′33″E	16-10-2019	Nymphs	Moderate
Al-Hajir (AHj)	23°23′30″N, 58°31′09″E	16-10-2019	Nymphs	High
Al-Wasil (AWl)	22°30′02″N, 58°44′41″E	18-10-2019	Nymphs	Moderate
Al-Kharma (AK)	22°51′11″N, 57°57′34″E	21-10-2019	Nymphs	Moderate
Miss (Ms)	23°08′13″N, 58°24′01″E	22-10-2019	Nymphs/Adults	Moderate
Ghiyazah (Gh)	23°07′22″N, 58°27′40″E	22-10-2019	Nymphs	High
Al-Ghabbi (AG)	22°26′29″N, 58°48′47″E	22-10-2019	Nymphs	High
Al-Ruddah (AR)	22°30′04″N, 58°07′04″E	22-10-2019	Nymphs/Adults	High
Baa’d (Bd)	23°05′48″N, 58°20′52″E	22-10-2019	Nymphs/Adults	Moderate
Hail Al-Khanabisha (HAK)	23°36′00″N, 56°32′13″E	23-10-2019	Nymphs	Moderate
Sint (St)	23°07′58″N, 57°05′22″E	23-10-2019	Nymphs/Adults	Moderate
Sayyah (Sh)	23°41′05″N, 56°33′31″E	23-10-2019	Nymphs	Moderate
Al-Hajar (AHr)	23°17′03″N, 56°55′18″E	23-10-2019	Nymphs	High
Al-Waqba (AWa)	23°34′18″N, 56°31′15″E	23-10-2019	Nymphs	Moderate
Al-Mudhairib (AM)	22°36′31″N, 58°40′07″E	25-10-2019	Nymphs	Moderate

### Insecticide formulations, solvent and enzyme assay kits

Plant Protection Research Center, Directorate General of Agriculture and Livestock Research, Al-Rumais, Oman provided the insecticide deltamethrin (Decis 2.5EC, Bayer Crop Sciences, Monheim am Rhein, Germany) and fenitrothion (Sumithion 50EC, Sumitomo Chemical, Tokyo, Japan) and acetone (> 90% purity; J.T. Baker, Avantor Performance Materials B.V. Teugseweg 20-7418AM Deventer, Netherlands) for using in the bioassays. The colorimetric enzyme assay kits (CPR activity, GST activity and AChE activity) were obtained from Abcam (Biomedical Scientific Services LLC, Al-Ain, Abu Dhabi, 69,480, United Arab Emirates), Cayman Chemical Company (Cayman Europe OÜ, Akadeemia tee 21/5, Tallinn, 12,618, Estonia) and BioAssay Systems, 3,191 Corporate Place, Hayward, CA 94,545, respectively.

### Experimental procedure

The residue film bioassay protocols were adapted to observe the toxicity of the selected insecticides (deltamethrin and fenitrothion) against different field collected populations of *O. lybicus*^51,52^. A measured volume (1 ml/petri plate) of each dilution (from range of dilutions determined through pre-bioassays and demonstrating 1–99% mortality of the test insect) was applied to the sterilized petri plates and the lid covers. A complete and even application of pesticide surface-residues were obtained by a continuous rotation of petri plates which were then set underneath the fume hood for evaporation. The petri plates in control treatment received acetone (without insecticide). Batches of each strain containing counted number of healthy and homogeneous 3rd instar nymphs were released in the pesticide-treated petri plates. After specified interval (48 h) of insect releases in the treated plates, mortality scores (dead insects) were noted separately and analyzed for comparison. The insects with least movement gestures (appendages) upon physical stimulations with the camel hairbrush were considered alive, and vice versa. The bioassays were performed with all necessary precautions and cares to diminish the experimental error. The data for mortality were corrected by using the abbot’s formula^53^.

$$\mathrm{Corrected\,Mortality }\left(\mathrm{\%}\right)=\left[1-\left\{\frac{\mathrm{n }\,(\mathrm{T\,after\,treatment})}{\mathrm{n \,}(\mathrm{Co\,after\,treatment}) }\right\}\right] {\times }100$$

where, n is number of insects, T is treated, and Co is Control.

The enzyme analyses were performed to detect the activities of CPR, GST and AChE in 3^rd^ instar nymphs of field collected, DEL-SEL and LAB-SUS strains of *O. lybicus*. The protocols provided by the manufacturer were strictly followed while performing the assays. Briefly, for CPR activity, whole body tissues (fresh samples) weighing 5 mg from each strain were homogenized in 500 µL of ice-cold CPR Assay Buffer after washing with Phosphate-buffered saline (PBS). After incubating for five minutes on ice, samples were centrifuged (1,500 × *g*) at 4 °C. The supernatant from each sample was transferred to pre-chilled microfuge tube and further centrifuged (12,000 × *g*) at 4 °C for 5 min. The supernatant was again transferred to pre-chilled tubes and stored on ice until CPR activity assay was performed. A standard curve was obtained from standard dilutions (0, 2, 4, 6, 8 and 10 nmol/well) of 1 mM Glucose-6-Phosphate (G6P). Samples of 5 µL G6P standard developer and 5 µL Nicotinamide adenine dinucleotide phosphate (NADPH) were added to the standard wells (containing 90 µL standard 1 mM G6P). After 30 min incubation at room temperature absorbance was measured at an optimal density of 460 nm. The 96-well plate was set up with samples (40 µL sample + 60 µL CPR assay buffer), samples + inhibitor (40 µL sample + 10 µL inhibitor + 50 µL CPR assay buffer), positive control (5 µL Recombinant CPR + 60 µL CPR assay buffer) and background wells (40 µL sample + 2 µL inhibitor + 58 µL CPR assay buffer). To each well, 30 µL of CPR Reaction Mix (5 µL NADPH substrate + 25 µL CPR assay buffer) was added. Finally, 10 µL of 20 mM G6P solution was added to each well except background wells. The absorbance was measured immediately at OD = 460 nm (25 °C) and continued until 30 min in a kinetic mode. The concentration of CPR in the test samples was calculated as

$$CPR\,Activity=\left(\frac{B}{\Delta T\times P}\right)={\rm (nmol/min)/mg}={\rm mU/mg}$$

where ‘B’ is the amount of G6P (nmol) consumed (calculated through the standard Curve), ‘∆T’ is the reaction time (minutes) and ‘P’ is the amount of protein sample (mg) added in the reaction well.

The whole-body tissues (3–5 mg) were used and rinsed with PBS (pH 7.4) for performing the GST activity assays. The tissues were then homogenized in 50 ml cold buffer (pH 7.0, containing 2 mM EDTA) and were centrifuged at 10,000 × *g* for 15 min (4 °C). The resultant supernatant was used in GST activity assays. The background wells were set up by adding 170 µL of Assay Buffer and 20 µL Glutathione. The positive control wells contained 150 µL of Assay buffer, 20 µL of Glutathione and 20 µL of reconstituted GST (control). In sample wells, we added 150 µL of Assay Buffer, 20 µL of Glutathione and 20 µL of samples. The reaction was initiated by adding 10 µL of CDNB (1-chloro-2,4-dinitrobenzene) to each well. The absorbance was measured once every minute at 340 nm at 5 time points. The GST activity was calculated as

$$GST\,\, Activity= \frac{\Delta {A}_{340}/min. }{0.00503\,\upmu {\rm {M}^{-1}}}\times \frac{0.02\,{\rm ml}}{0.002\,{\rm ml}} \times Sampl\mathrm{e}\,dilution = {\rm nmol/min/ml}$$

where ‘∆A_340_′ is the change in absorbance per minute and was determined as

$$\Delta {A}_{340}/\mathrm{min}= \frac{{A}_{340} \left(Time 2\right)-{A}_{340} (Time 1)}{Time 2 \left(min.\right)-Time 1 (min.)}$$

In order to perform the AChE activity assays, supernatant was obtained from homogenization of whole-body tissues (3–5 mg) in 0.1 M phosphate buffer (pH 7.5) and 5 min centrifugation at 14,000 rpm. Briefly, 2 mg reagent was mixed in 200 μL Assay Buffer to prepare the Working Reagent. A volume (200 μL) of each, water and calibrator, were added separately to the wells, along with samples (10 μL) in separate wells. The reaction was initiated by transferring freshly prepared Working Reagent (190 μL) to all sample wells and the plats were tapped briefly to mix well. First absorbance was measured at OD = 412 nm at 2 min and second at 10 min in the plate reader. AChE activity was calculated as follows,

$$AChE\, Activity= \frac{{OD}_{10} - {OD}_{2}}{{OD}_{CAL} - {OD}_{H2O}} \times 200 (U/L)$$

where ‘OD_10_′ and ‘OD_2_′ are the OD_412nm_ values of samples at 10 min and 2 min, respectively. ‘OD_CAL_’ and ‘OD_H2O_’ are the OD_412nm_ values of Calibrator and water at 10 min. The number ‘200′ is the equivalent activity of the calibrator under assay conditions.

### Data analyses

The toxic dose values (LD_50_) of deltamethrin for test populations, resistance ratios over susceptible (Reference) strain and their confidence interval values were estimated by performing the Probit Analysis^54^ of the recorded mortalities in each experiment using Polo Plus software version 1.0 (LeOra software LLC). Treatments with non-overlapping confidence intervals were considered significantly different for their toxicities (LD_50_). Higher slope numbers (> 0.90) described lower dissimilarity in physical appearances of the treated insects from each field strain^55^. The resistance ratios (RRs) were calculated through the division of median lethal dose of the respective (field/selected) strain and median lethal dose the reference strain. Categories for resistance were determined by following the scale described by Jin and his co-workers^56^ i–e susceptible (RR < 3 folds), minor resistance (3 ≤ RR ≥ 5 folds), low resistance (5 ≤ RR ≥ 10 folds), intermediate resistance (10 ≤ RR ≥ 40 folds), high resistance (40 ≤ RR ≥ 160 folds) and extremely high resistance (RR > 160 folds). The values of RRs were measured significantly different only when 95% CI (confidence interval) did not include 1 ^54^. The results obtained in enzyme activity studies were analyzed for variance and the means were compared by Tukey’s HSD test through Minitab 18 software.

## Ethical statement

Any author did not perform any experiment with humans as participants. All the standard protocols for performing the bioassay and enzyme activity experiments with the insect *O. lybicus* belonging to phylum Arthropoda, were performed after approval from the Plant Protection Research Center, Ministry of Agriculture and Fisheries, Oman.

## Availability of data and materials

All observations recorded or procured during the experiments are submitted to the Plant Protection Research Center, Directorate General of Agriculture and Livestock Research, Ministry of Agriculture and Fisheries, Oman and The Research Council (TRC), Oman, however, statistically analyzed data are presented in the manuscript. The data can be only available upon the consent of the concerned authorities.
